# Processability of mesoporous materials in fused deposition modeling for drug delivery of a model thermolabile drug

**DOI:** 10.1016/j.ijpx.2022.100149

**Published:** 2022-12-17

**Authors:** Christos S. Katsiotis, Maria Strømme, Ken Welch

**Affiliations:** Division of Nanotechnology and Functional Materials, Department of Materials Science and Engineering, Uppsala University, Box 35, Uppsala SE-751 03, Sweden

**Keywords:** FDM, Hot-melt extrusion, Experimental design, Poorly-soluble drug, Mesoporous material, Thermal protection

## Abstract

The incorporation of drug-loaded mesoporous materials in dosage forms prepared with fused deposition modeling (FDM) has shown the potential to solve challenges relating to additive manufacturing techniques, such as the stability of poorly-soluble drugs in the amorphous state. However, the addition of these non-melting mesoporous materials significantly affects the mechanical properties of the filament used in FDM, which in turn affects the printability of the feedstock material. Therefore, in this study a full-factorial experimental design was utilized to investigate different processing parameters of the hot melt extrusion process, their effect on various mechanical properties and the potential correlation with the filaments' printability. The thermolabile, poorly-soluble drug ibuprofen was utilized as a model drug to assess the potential of two mesoporous materials, Mesoporous Magnesium Carbonate (MMC) and a silica-based material (MCM-41), to thermally protect the loaded drug. Factorial and principal components analysis displayed a correlation between non-printable MCM-41 filaments and their mechanical properties where printable filaments had a maximum stress >7.5 MPa and a Young's modulus >83 MPa. For MMC samples there was no clear correlation, which was in large part attributed to the filaments' inconsistencies and imperfections. Finally, both mesoporous materials displayed a thermal protective feature, as the decomposition due to the thermal degradation of a significant portion of the thermolabile drug was shifted to higher temperatures post-loading. This highlights the potential capability of such a system to be implemented for thermosensitive drugs in FDM applications.

## Graphical abstract

**Figure:**
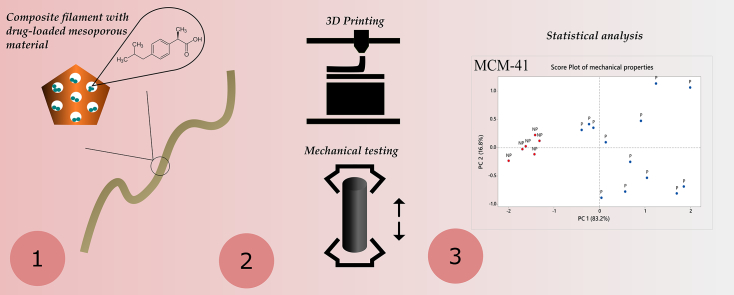

## 1 Introduction

Applications of Additive Manufacturing (AM), both in everyday life and various research fields, have significantly increased in the past decade. In the pharmaceutical field specifically, AM can serve as a means to modernize and advance drug development and delivery (Mathew et al., 2020). Among the various AM techniques, Fused Deposition Modeling (FDM) is the one that has been investigated the most in the pharmaceutical field. The feedstock material for FDM is a polymeric filament, which can be obtained commercially or developed in the laboratory. For drug delivery applications the drug is usually embedded in the filament. This is achieved either by soaking a pre-prepared filament in a solution containing the drug, or, most commonly, via Hot Melt Extrusion (HME) (Tagami et al., 2019; Gioumouxouzis et al., 2018). Custom filaments produced with HME are achieved by mixing the drug with the polymer and other additives, and extruding the mixture at elevated temperatures through a die with the aid of a screw.

Although FDM offers a fast and facile means of developing patient-tailored drug formulations, there are also several challenges involved with its use. For example, since a relatively high temperature is normally used with FDM and HME, thermolabile Active Pharmaceutical Ingredients (APIs) are excluded from being used directly with these methods (Eleftheriadis et al., 2020a). As more thermosensitive drugs become available and increase their share in the overall market, different ways of implementing them in 3D printed formulations have been employed. In general, semi-solid extrusion has been the predominant technique, as it is applied with a considerably lower temperature compared to FDM (Abdella et al., 2021). For formulations produced with FDM, choosing a polymer with a lower T_g_, and consequently lower processing temperature, is a commonly used approach to address this issue (Kempin et al., 2018; Okwuosa et al., 2016). Additionally, different drug delivery systems have been developed where the thermolabile ingredient is introduced via filling an FDM produced container, thus avoiding the high temperature needed for the print (Eleftheriadis et al., 2020b; Cotabarren and Gallo, 2020). Other challenges involved with employing FDM for producing drug formulations are the recrystallization of the drug in the solid dispersions of the filaments and issues with the integrity of the polymeric filament (Solanki et al., 2018; Macedo et al., 2020; Okwuosa et al., 2021). Furthermore, incorporating poorly soluble drugs is a challenge for FDM-produced dosage forms, although this challenge is not unique to the FDM technique. A great proportion of newly developed drugs are poorly soluble (Ibrahim et al., 2020), and for them to achieve an efficient dissolution profile, which is a necessary precondition for bioavailability, they need to be in the amorphous state. One of the ways to achieve this is through a solid dispersion system, such as a hot melt extruded filament. However, as previously mentioned, the long-term stability issue of such systems would lead to the recrystallization of the drug and therefore result in a suboptimal drug release upon dissolution.

An approach for addressing the aforementioned challenges is to incorporate mesoporous materials in the dosage forms. Suitable mesoporous materials have a large surface area and narrow pores that can restrict the recrystallization of drug molecules that are incorporated in them (Bremmell and Prestidge, 2019). Mesoporous silica, specifically MCM-41, has been used extensively for such applications, either as synthesized or after post synthesis modifications. It has a hexagonal structure and, depending on the synthesis route, a pore width range between 1.5 and 10 nm (Muñoz et al., 2003; Manzano et al., 2008). Another mesoporous material, mesoporous magnesium carbonate (MMC), is a recently developed material that can be synthesized via a template-free route. Depending on the synthesis parameters, its properties can be tuned according to the application's needs, i.e., with a specific surface area ∼ 300–800 cm^2^/g and pore widths from 2 to 20 nm (Cheung et al., 2016; Zhang et al., 2016; Zhang et al., 2014; Forsgren et al., 2013). Both materials, when drug-loaded, have been shown to be chemically stable for a long period of time, retaining the drug in the amorphous state (Alvebratt et al., 2020; Bukara et al., 2016). Recently, it has been demonstrated that these mesoporous materials can be loaded with a poorly soluble drug and incorporated in composite filaments for FDM of novel dosage forms, which exhibit desirable drug release behaviors. (Katsiotis et al., 2021). Furthermore, it should be noted that thermostability issues arising with the use of HME and FDM techniques can be addressed with the aid of mesoporous materials. Examination of data from thermogravimetric analyses in previous studies shows that drugs loaded in the pore systems of various mesoporous materials exhibit a delay in thermal degradation compared to the drugs' free forms, which suggests a thermal protection effect (Zhang et al., 2016; Katsiotis et al., 2021; Abbaraju et al., 2014; Christoforidou et al., 1896).

Although the incorporation of mesoporous materials offers a potential solution to several of the issues involved with FDM of dosage forms, it also introduces an additional challenge, namely the printability of the resulting filaments. An important limiting aspect in FDM generally is the printability of the produced filaments, and as such, this aspect has been the focus of several studies (Öblom et al., 2019; Nasereddin et al., 2018; Elbadawi et al., 2020). The term “printability” itself has no exact definition and strongly depends on the researchers' experience. Generally, it refers to the ability of the filament to be directly fed to the printer's head without breaking or bending. Parameters such as the filament's viscosity and mechanical properties, as well as the filament components' miscibility have been found to be correlated with the printability of the filament, where mechanical properties are the most commonly investigated parameters (Elbadawi et al., 2020; Alhijjaj et al., 2016; Gioumouxouzis et al., 2020; Tabriz et al., 2021). The introduction of non-melting components such as mesoporous materials in the feedstock can negatively affect printability, particularly in high proportions compared to the thermoplastic components. Consequently, towards a generalization of the strategy of employing mesoporous materials in FDM feedstock, an investigation of the printability of the composite filaments is of great interest. Therefore, in this study four factors in the extrusion process were investigated to determine their effect on the mechanical properties of the filament and if they can be correlated to the printability of the filament. A full factorial experimental design was implemented with the following four factors: the degree of drug loading in the mesoporous material; the percentage of mesoporous material in the mixture; the plasticizer to polymer ratio; and the extrusion temperature. Furthermore, ibuprofen was employed as a model thermolabile, poorly soluble drug to emphasize the potential advantage of such systems.

## 2 Materials and methods

### 2.1 Materials

Mesoporous magnesium carbonate (MMC) with a particle size <100 μm was kindly provided by Disruptive Materials AB (Uppsala, Sweden). In order to remove any organic residuals from synthesis, the received material was heat-treated in a furnace with a 10 h ramp from room temperature to 250 °C, with an addition 10 h hold at this temperature. Ibuprofen was purchased from Toronto Research Chemicals (Toronto, Canada). d-mannitol, ethanol absolute >99.8%, ammonia 28%, and tetraethyl orthosilicate 98% (TEOS) were purchased from VWR International (Stockholm, Sweden). Hexadecyltrimethylammonium bromide (CTAB) and polyvinyl alcohol 4–88 (PVA) were both purchased from Sigma-Aldrich (Stockholm, Sweden).

### 2.2 Synthesis of MCM-41

MCM-41 was prepared using an adjusted protocol based on a synthesis previously reported (Katsiotis et al., 2021). First CTAB was added to water and mixed with ammonia. TEOS was subsequently added to the mixture dropwise at room temperature. After 1 h of stirring the mixture was filtered and washed with water. The solid residue was dried overnight at 90 °C. Finally, the removal of the organic template was achieved by calcination in a furnace where the temperature was raised from room temperature to 550 °C at 1 °C /min and held at this temperature for 5 h. The molar ratios for the components in the synthesis were 1 TEOS: 0.152 CTAB: 2.8 NH_3_: 141.2 H_2_O.

### 2.3 Experimental design

In order to assess the effect of the selected factors on the mechanical properties of the filaments, a full factorial design for each mesoporous material was applied using Minitab software (version 17.1, Minitab, LLC, State College, PA, USA). For both MMC and MCM-41 the same factors were studied, but as some of the factors' maxima differed between the two materials, a separate factorial experiment was used for each material. The factors studied were the following: (A) % Drug Loading, referring to the percentage of available pore volume in the mesoporous material that is loaded with ibuprofen, (B) % Mesoporous material content, referring to the percentage of loaded or unloaded mesoporous material within the mixture to be extruded, (C) Plasticizer/Polymer, referring to the ratio of plasticizer to polymer within the mixture to be extruded, and (D) Temperature, referring to the extrusion temperature. Preliminary experiments were performed to determine the low and/or high values for each factor in each factorial experiment. For % Drug Loading, the low point was set at 0% and the high point was determined by increasing the drug loading until evidence of drug crystallinity in the mesoporous materials was observed. For % Mesoporous material content, the low point was set at 10% for both materials as to have enough in the mixture to produce an observable effect on the mechanical properties while the high point was limited by the level beyond which the mixture was not extrudable. For the Plasticizer/Polymer ratio, the high point was set at 1 so that the polymer would not be less than the plasticizer in the mixture. The low setting was determined by the least amount of plasticizer needed for the mixture to be extrudable when the mesoporous material content was at its high point. Finally, initial extrusions were conducted with the loaded and unloaded materials to test for the lowest temperature that a mixture is extrudable. The high point was then set at a few tens of degrees higher to study the effect of the factor but not be too warm such that mixture's components might degrade. The values for the factors for each material are shown in Table 1. The response variables in the design were the Young's modulus and tensile strength of extruded filaments, as well as the printability of the filaments.

**Table 1:** Factor values used in the factorial experiments for the two mesoporous materials MMC and MCM-41.

	**MMC**			**MCM-41**		
**Factor**	**Low**	**High**	**Midpoint**	**Low**	**High**	**Midpoint**
(A) % Drug Loading	0	90	45	0	80	40
(B) % Mesoporous material content	10	40	25	10	35	22.5
(C) Plasticizer/Polymer	0.4	1	0.7	0.4	1	0.7
(D) Temperature (°C)	160	190	175	160	190	175

### 2.4 Drug loading of the mesoporous materials

The solvent evaporation method was used to load the drug in the two mesoporous materials. The target loadings were selected based on the experimental design and the preliminary tests and they refer to the percentage of available pore volume filled with the drug. For MCM-41 the target loadings were 80% and 40%, whereas for MMC they were 90% and 45% of their respective empty pore volume. Based on the theoretical molar volume of ibuprofen and the empty pore volume of each mesoporous material measured via gas sorption analysis, the masses used for loading were as following. For MMC 90%, 17.5 g of MMC were added to a solution of 7.58 g ibuprofen in 500 mL ethanol. For MMC 45%, 12 g MMC was added to a solution of 2.56 g ibuprofen in 500 mL ethanol. Accordingly, for MCM-41 80%, 16 g of MCM-41 was added to a solution of 10.4 g ibuprofen in 500 mL of ethanol, and for MCM-41 40%, 8 g of mesoporous material was added to a solution of 2.6 g ibuprofen in 500 mL ethanol. All these mixtures were directly placed in a rotary evaporator until the solvent was removed. The solid residue of each formulation was subsequently dried in an oven overnight at 70 °C. The confirmation of the drug loading percentages was done via gas sorption and thermogravimetric analysis.

### 2.5 Gas sorption analysis

This analysis was performed with an ASAP 2020 (Micromeritics Instrument Corporation, Norcross, GA, USA). Isotherms of the samples were collected at liquid nitrogen temperature (−196 °C) over a relative pressure range (p/p^0^) of 0–1. The samples were first degassed using a Micromeritics Smart VacPrep unit under dynamic vacuum (1 × 10^−4^ Pa) for 16 h at 70 °C. Specific surface area (SSA), pore size distribution, and total pore volume were calculated using MicroActive version 5 software (Micromeritics, Norcross, GA, USA). SSA was calculated using the Brunauer–Emmett–Teller (BET) model over the relative pressure range of 0.05–0.25. The pore size distribution was calculated using density functional theory (DFT) to analyze the nitrogen sorption isotherms. Finally, total pore volume was measured through a single-point adsorption at a relative pressure of p/p^0^ = 0.985.

### 2.6 Hot melt extrusion (HME)

The filaments for the factorial experiments were prepared via HME using a Filabot EX2 single screw extruder (Filabot Inc., Barre, VT, USA) with a tunable screw speed between 0 and 35 rpm. PVA pellets were first ground to smaller particles, sieved through a 1 mm mesh, and then mixed in a mortar with the plasticizer d-mannitol and mesoporous material powder used in the extrusion. Each filament was produced from 15 g of powder mixture and extruded through a 1.5 mm diameter nozzle with screw speed of 12 rpm. All filaments produced were collected and stored in air-tight sealed plastic bags until analysis. The values of the four factors used to make the filament in each experimental run are shown in Table 2. Additionally, the percentage composition of these filaments can be found in Table S1.

**Table 2:** Values of the four factors in each experimental run (Run Order) for the MMC factorial experiment and the MCM-41 factorial experiment.

	**MMC**				**MCM-41**			
Run Order	% Drug Loading	% Meso- porous	Plasticizer/ Polymer	Temp (°C)	% Drug Loading	% Meso- porous	Plasticizer/Polymer	Temp (°C)
1	0	40	0.4	190	0	35.0	1.0	160
2	90	40	0.4	190	40	22.5	0.7	175
3	45	25	0.7	175	80	10.0	0.4	190
4	0	40	1.0	190	0	10.0	0.4	160
5	0	10	0.4	190	80	35.0	1.0	160
6	90	10	0.4	160	0	35.0	0.4	190
7	0	10	0.4	160	80	10.0	0.4	160
8	0	40	0.4	160	80	10.0	1.0	160
9	90	40	0.4	160	40	22.5	0.7	175
10	90	10	1.0	160	0	10.0	0.4	190
11	0	40	1.0	160	80	35.0	0.4	160
12	90	10	0.4	190	40	22.5	0.7	175
13	90	10	1.0	190	80	35.0	1.0	190
14	0	10	1.0	160	0	35.0	0.4	160
15	45	25	0.7	175	0	35.0	1.0	190
16	90	40	1.0	190	0	10.0	1.0	190
17	90	40	1.0	160	0	10.0	1.0	160
18	45	25	0.7	175	80	10.0	1.0	190
19	0	10	1.0	190	80	35.0	0.4	190

### 2.7 Attenuated total reflectance–fourier-transform infrared spectroscopy (ATR–FTIR)

ATR–FTIR spectra of all samples were recorded using a Tensor 27 spectrometer (Bruker, Billerica, MA, USA) together with a platinum ATR diamond module (Bruker, Billerica, MA, USA). The spectra were recorded in the range of 400–4000 cm^−1^ at 4 cm^−1^ resolution with 128 scans.

### 2.8 Differential scanning calorimetry (DSC)

DSC measurements were performed on samples of approximately 5 mg in perforated aluminum crucibles using a DSC 3+ instrument (Mettler Toledo, Schwerzenbach, Switzerland). Samples were cooled from room temperature to −35 °C and then heated to 300 °C at a rate of 5 °C/min.

### 2.9 Thermogravimetric analysis (TGA)

TGA measurements were performed with a TGA/DSC 3+ (Mettler Toledo, Schwerzenbach, Switzerland). Samples of approximately 20 mg were loaded in uncovered alumina pans and heated from room temperature to 700 °C at a rate of 10 °C /min in air atmosphere. A second protocol was also followed for some samples. The loaded alumina pans were heated from room temperature to 150 °C at a rate of 10 °C /min. After that a cycle of isothermal holding for 20 min and dynamic heating of 10 °C /min was followed every 10 °C until 250 °C. Subsequently they were heated to 700 °C in air atmosphere.

### 2.10 Printability assessment

Printability testing was performed using a Prusa i3 MK3S printer (Prusa Research a.s., Prague, Czech Republic). In order to evaluate the printability of the filaments developed, an empirical assessment was employed in which filaments were loaded into the printer and extruded in a line for approximately 20 cm. Filaments that could be successfully loaded and extruded would be rated printable, whereas filaments that cracked or could not be loaded would be rated as unprintable. Printer temperatures for the loading of the filaments ranged between 190 and 205 °C.

### 2.11 Scanning electron microscopy (SEM)

The morphology of the two mesoporous materials MMC and MCM-41 was studied with SEM. Samples were first sputter-coated with Au/Pd and subsequently imaged with a LEO 1550 SEM (Zeiss, Jena, Germany) at an acceleration voltage of 5.0 kV.

### 2.12 Mechanical studies

The Young's modulus and tensile strength of the produced filaments were the mechanical properties chosen as response variables in the two-level-full factorial experimental design, and were assessed using a universal testing instrument (AGS-X, Shimadzu, Kyoto, Japan). Three sections of each filament sample were tested. Each section was 12 cm long where the end 2 cm of the section was held by grippers, resulting in a gauge length of 8 cm. A strain rate of 1 mm/s was used in the mechanical testing (Tabriz et al., 2021). The diameter of each specimen was measured with a digital caliper at several points along its length, and the average value was used for the analysis.

### 2.13 Factorial analysis and principal components analysis (PCA)

Statistical analyses of the factorial experiments were conducted on the data collected from the mechanical studies. The main effects and two-way interactions of the factors were examined in relation to the response variables Young's modulus and tensile strength. Moreover, a Principal Component Analysis (PCA) was performed on the same data. Two principal components (PCs) were extracted and used to construct a score plot of the samples of each mesoporous material by applying a singular vector decomposition. The above analyses were run on the Minitab software.

## 3 Results and discussion

### 3.1 Particle size and morphology assessment

**Fig. 1:**
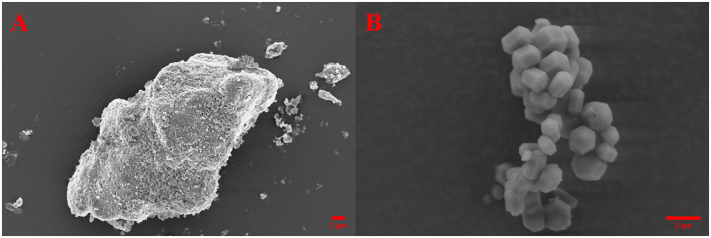
SEM images of mesoporous materials (A) MMC, (B) MCM-41.

The morphology of the two mesoporous materials was assessed with SEM imaging. MMC particles can vary in size and shape as they are formed by the aggregation of smaller nanoparticles (Cheung et al., 2016), whereas the template-based synthesis route of MCM-41 leads to more uniform primary, see Fig. 1. The MMC aggregates range in size from <1 μm to several tens of μm and appear to have a rough surface. The primary particles of MCM-41 are generally between 1 and 2 μm and exhibit a hexagonal geometry as a result of the organic template used during synthesis. The morphology of both mesoporous materials used in this study has previously been shown to be unaffected by the drug loading process (Katsiotis et al., 2021).

### 3.2 Gas sorption measurements

Nitrogen gas sorption measurements were conducted on the two mesoporous materials MMC and MCM-41 with their different degrees of ibuprofen loading (0%, 45% and 90% of available pore volume with MMC versus 0%, 40% and 80% of available pore volume with MCM-41). In Figs. S1-S2 the desorption and adsorption isotherms for the samples are shown, along with their pore size distributions. All samples exhibit a typical type IV shape, in accordance with previous reports (Brunauer et al., 1940; Yang et al., 2017; Tzankova et al., 2019). Table 3 presents the specific surface area (SSA), pore volume and pore width of the samples. For both types of mesoporous materials, the SSA and pore volume are reduced after the ibuprofen loading. A similar trend is observed with the pore size distributions where the pore size (width) for the mesoporous materials decreases with increased ibuprofen loading.

**Table 3:** SSA, pore volume and pore width for unloaded and drug-loaded MMC and MCM-41.

Sample	SSA m^2^/g	Pore volume cm^3^/g	Pore width
MMC unloaded	328.9	0.45	5.43
MMC Ibuprofen 45%	220.0	0.22	4.66
MMC Ibuprofen 90%	74.7	0.04	3.43
MCM-41 unloaded	1101.3	0.77	3.18
MCM-41 Ibuprofen 40%	729.9	0.37	2.52
MCM-41 Ibuprofen 80%	357.8	0.20	2.34

In order to achieve the desired ibuprofen loadings for MMC and MCM-41, a targeted loading approach was employed. First, the pore volume of the unloaded MMC and MCM-41 was calculated to be 0.45 cm^3^/g and 0.77 cm^3^/g, respectively, shown in Table 3. The predicted molar volume of ibuprofen of 200.3 cm^3^ (*Ibuprofen | C13H18O2 | ChemSpider Available online*, n.d.) was used to calculate the mass of ibuprofen required to achieve the desired loadings. A maximum loading target of 90% *v*/v was initially selected to ensure that all of the drug would be incorporated into the pores in the amorphous state, without having some form crystals on the outer surface. Using the solvent evaporation method of loading the drug into the pores also removes the need for a washing step after drying, as would be required if a different drug loading approach was utilized (Seljak et al., 2020; Trzeciak et al., 2021). While a theoretical target loading of 90% *v*/v ibuprofen could be achieved with MMC, which was validated both by gas sorption and DSC measurements, it was not possible with MCM-41, and thus the maximum theoretical loading for MCM-41 was reduced to 80%. It is possible that capillary condensation of drug molecules in the pores closer to the surface resulted in some pore volume being blocked from complete loading. Another reason for the different loading behaviors between the two materials could be attributed to their differences in surface chemistry and/or different widths of their pores, allowing for different interactions between the mesoporous materials and ibuprofen.

The pore volume measurements from Table 3 were used to calculate the actual drug loadings in both *v*/v % and *w*/w %, and the comparison to the theoretical loadings is displayed in Table 4. The results show that this approach of targeted drug loading is a viable one, with good agreement between the theoretical and measured loading levels.

**Table 4:** Drug-loading percentage *v*/v % and *w*/w % in theory and calculated via gas sorption measurements, for MMC and MCM-41 samples.

Sample	Theoretical loading		Measured loading	
	v/v %	w/w %	v/v ± sd%	w/w ± sd %
MMC Ibuprofen 45%	45%	16.3%	50 ± 2%	18.8 ± 0.5%
MMC Ibuprofen 90%	90%	29.4%	91.3 ± 0.5%	29.7 ± 0.1%
MCM-41 Ibuprofen 40%	40%	24.1%	52 ± 3%	29 ± 1%
MCM-41 Ibuprofen 80%	80%	38.8%	74 ± 2%	36.9 ± 0.5%

### 3.3 Filament extrusion

A common observation in HME of PVA formulations is die swell, leading to filaments with bigger diameters than the nozzle used (Wang, 2012). Hence, a 1.5 mm diameter nozzle was used during the process, which produced filament diameters ranging from 1.5 up to 1.8 mm. Example images of filaments can be seen in Fig. S3. It has been previously noted that the addition of non-melting components, i.e., MMC and MCM-41, in the HME process results in formulations that are solid dispersions of mesoporous particles within the polymer and plasticizer blend (Katsiotis et al., 2021). The addition of these components can cause the filament to become brittle and hence not printable. By varying the extrusion temperature and ratios of mesoporous material, polymer and plasticizer, this effect can be mitigated, however, a poor choice of the aforementioned parameters, e.g., excessively high content of non-melting material or low extrusion temperature, can result in the mixture not being extrudable. The physical mixture in such cases tends to become compressed prior to reaching the extruder nozzle and clogs the barrel due to the high pressures arising within it. To avoid such cases in the factorial experiments, initial testing revealed a range of temperatures and powder mixture ratios that would result in extrudable formulations (data not shown), and these data were used to set the factor values used in the factorial experiments, see Table 2. Understandably, some settings resulted in better filaments than others, and in some cases, the filaments were difficult to extrude in a single, continuous piece, indicating that these formulations would also be unprintable.

### 3.4 FTIR

Spectra of ibuprofen and the individual component powders of the filaments, as well as one filament sample per mesoporous material, are presented in Fig. S4. The filament samples chosen are the ones having with highest drug loading for each mesoporous material. Ibuprofen exhibits bands in the regions of 2954, 1708, 1510 and 1230 cm^−1^ corresponding to C—H, C=O stretching, C—C ring vibrations and C—O stretching / O—H bending, respectively (Namur et al., 2009). The spectrum for PVA exhibits characteristic peaks at 3330, 2910–2940, 1714–1734, 1425, 1083, and 842 cm^−1^ due to O—H stretching, C—H stretching, C=O stretching, CH_2_ bending, C—O stretching, and C—C stretching, respectively (Kharazmi et al., 2015; Bhat et al., 2005). The characteristic peaks in mannitol's spectrum agree with previous reports (Al-khattawi et al., 2014; Burger et al., 2000). In the spectrum of unloaded MMC, bands in the regions of 1442, 1085 and 852 cm^−1^ corresponding to the carbonate groups appear (Cheung et al., 2016; Frykstrand et al., 2014). Unloaded MCM-41 displays peaks at 802 and 1062 cm^−1^ due to Si—O stretching and Si—O—Si vibrations, respectively (Guo et al., 2015). The drug-loaded samples of MMC Ibuprofen 45% and MMC Ibuprofen 90% present no significant differences compared to the pure MMC. MCM-41 Ibuprofen 40% and − 80% powders exhibit similar spectra to the one of unloaded MCM-41, with a small extra peak at 1708 cm^−1^ present, attributed to C=O stretching of the —COOH group of ibuprofen. The various peaks in the spectra of both filament samples can be explained by superposition of constituent peaks and present no unexpected appearance or disappearance of peaks.

### 3.5 DSC

**Fig. 2:**
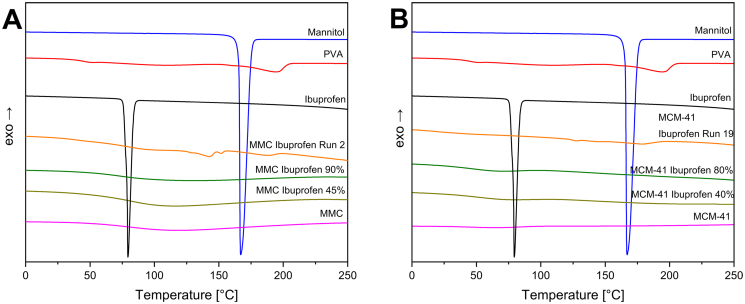
DSC thermograms of ibuprofen, the individual component powders of the filaments, and sample filaments MMC Run 2 and MCM-41 Run 19 for (A) MMC-containing samples, and (B) MCM-41-containing samples.

DSC measurements were primarily used to check for the presence of crystallinity of the drug after it was loaded in various degrees in the two mesoporous materials and to investigate potential material transitions within the temperature range of −35 to 300 °C. Fig. 2 depicts the thermograms of ibuprofen and the individual component powders of the filaments, as well as one filament sample per mesoporous material. The filament samples displayed in Fig. 2 are the ones with the highest degree of drug loading, highest percentage of the mesoporous material in the mixture, lowest ratio of plasticizer to polymer, and highest extrusion temperature, i.e., 190 °C.

The DSC curves of mannitol and ibuprofen present sharp endothermic peaks at 167 °C and 80 °C, respectively, which corresponds to the literature values for their melting points (Al-khattawi et al., 2014; Eleftheriadis et al., 2020c). PVA exhibits a shift in the baseline in the range 50–60 °C, associated with its glass transition, and an endothermic process starting at 160 °C relating to its melting point (Pshezhetskii et al., 1990). As expected, MMC and MCM-41 exhibit no significant thermal events in the measured temperature interval. In the case of MMC there is only a broad endotherm in the range 50 to 120 °C due to evaporation of strongly bound moisture. Most significantly, the ibuprofen loaded samples MMC Ibuprofen 45% and − 90%, and MCM-41 Ibuprofen 40% and − 80%, present no sharp endothermic peak associated with the melting point of ibuprofen, providing a strong indication of successful loading and amorphization of the drug within the pore system of each material. This peak was also absent in the DSC curves of the filament samples, indicating that there was no recrystallization of ibuprofen due to the extrusion process. However, a depression of the melting point of PVA due to the presence of the plasticizer mannitol in the filament samples was observed. It should also be noted that the ability of MMC and MCM-41 to maintain drugs such as ibuprofen and celecoxib loaded within their pores in the amorphous state has previously been confirmed by DSC and XRD measurements (Zhang et al., 2016; Zhang et al., 2014; Katsiotis et al., 2021).

### 3.6 TGA

**Fig. 3:**
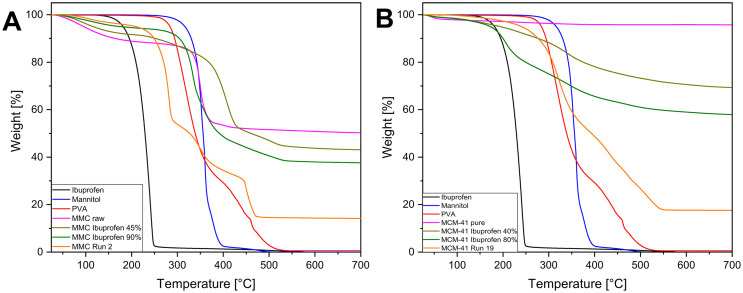
TGA thermograms of ibuprofen, the individual component powders of the filaments and sample filaments MMC Run 2 and MCM-41 Run 19 for (A) MMC-containing samples, and (B) MCM-41-containing samples.

The various raw materials and formulations were analyzed via TGA to investigate their thermal decomposition over the range of temperatures from 25 to 700 °C. Fig. 3 depicts the thermograms for ibuprofen, the individual component powders of the filaments, and one filament sample for each mesoporous material. For better visual clarity, only the filament sample with the highest drug loading is shown. Ibuprofen presented a one-step degradation process with an onset at ∼170 °C, whereas PVA started rapidly decomposing at ∼270 °C. Mannitol was shown to start its decomposition after 285 °C. For MMC, there is an initial decrease in mass of ∼11% attributed to water evaporation from the material's pore system. Subsequently, at ∼330 °C the decarbonation process begins, leaving only MgO at the highest temperatures reached in the measurement (Zhang et al., 2014). In contrast, MCM-41 remains unaffected by the heat-treatment up to 700 °C. There is, however, a slight mass loss ∼4% in the beginning of the process due to moisture desorption.

It has been previously observed that loading a drug in the pore system of mesoporous materials can delay the drug's decomposition compared to the pure crystalline drug, effectively increasing its thermal stability (Katsiotis et al., 2021). This behavior was also observed in both mesoporous materials used in the present study. The MMC Ibuprofen 45% sample showed a step in its thermogram with an onset of ∼260 °C, attributed to the decomposition of ibuprofen, whereas for MMC Ibuprofen 90% the drug decomposition appears to coincide with the decarbonation step at ∼290 °C. For both these samples the onset of the drug decomposition has been increased by 90 °C and 120 °C, respectively, indicating the thermal protection properties of MMC. This thermal protection behavior is also present in the case of MCM-41 drug-loaded formulations, although the behavior is somewhat different. The onset of ibuprofen degradation in the drug-loaded MCM-41 samples begins at approximately the same temperature as with the pure drug, but a significantly higher percentage of ibuprofen remains in the loaded samples at higher temperatures compared to the pure drug, see Fig. 3B. Of the two loaded samples, this shift to higher temperatures is more pronounced for the MCM-41 Ibuprofen 40% sample. This behavior could be explained by the position of the loaded drug within the pores. Drug that is located near the entrance to the pores may experience less thermal protection than drug located deeper within the pore structure. In the MCM-41 Ibuprofen 80% sample, the pores are filled to a greater extent than with the MCM-41 Ibuprofen 40% sample, and thus a larger proportion of the loaded drug would be located closer to the pore entrance. However, another possibility for the observed thermal protection effects could simply be due to the time it takes for degradation products to desorb and diffuse from within the porous structure. In order to check for slow diffusion effects, another TGA experiment was run where the temperature was increased in step intervals of 10 °C and held at these temperatures to allow time for degradation products to leave the pores. Fig. S5 in supplementary information displays the results from this experiment where it can be seen that the loading of the drug within the pores does provide a thermal protection and this effect is not a result of a slow diffusion of degradation products. While all the drug loaded in the MCM-41 Ibuprofen 40% experiences some degree of thermal protection, approximately 20% of the drug in the MCM-41 Ibuprofen 80% sample does not seem to be thermally protected as the first part of the curve follows the degradation curve of the crystalline Ibuprofen (see Fig. S5 B).

Overall when comparing the MCC versus MCM-41formulations, the degree of thermal protection appears to be greater for the MMC formulations. Furthermore, in the MMC formulations the protection is greater for the higher degree of drug loading, whereas in the MCM-41 formulations the protection is greater for the lower drug loading level. These differences in thermal protection behavior between the two mesoporous materials may be due to differences in pore size, shape and distribution, or interactions between the drug molecules and the mesoporous materials.

The TGA analysis can also be used to calculate the drug loading indirectly for each of the formulations. As stated above, MMC decomposes into CO_2_ and MgO during heating. The ratio between the remaining MgO and the initial MMC weight percentages can be compared to the drug-loaded MMC in order to calculate the drug content, as previously described (Katsiotis et al., 2021). A similar analysis of the TGA thermograms can be used to determine content of MMC in the filament. As for the drug-loaded MCM-41 powder, the calculation of the drug content is straightforward as the sole mass loss is attributed to the loaded ibuprofen, after accounting for any moisture desorption. Likewise, the MCM-41 content in the filament formulations can be extracted knowing that only MCM-41 remains at the highest temperatures during the measurement. Table 5 presents the drug loading *w*/w % determined by TGA measurements compared to the theoretical values for each mesoporous material. The values determined by TGA measurements are in good agreement with the theoretical values, and also reaffirm the values calculated by the gas sorption measurements. Tables S2 and S3 show the percentage of mesoporous material in the filaments of the 19 experimental runs for MMC and MCM-41 containing materials, respectively, calculated from the TGA traces.

**Table 5:** Drug loading percentage *w*/w in theory and calculated via TGA for MMC and MCM-41 samples.

Sample	Theoretical loading w/w %	Measured loading w/w ± sd %
MMC Ibuprofen 45%	16.3%	13.9 ± 1.1%
MMC Ibuprofen 90%	29.4%	27.0 ± 0.8%
MCM-41 Ibuprofen 40%	24.1%	26.3 ± 0.2%
MCM-41 Ibuprofen 80%	38.8%	37.6 ± 0.7%

### 3.7 Printability assessment

The printability of the produced filaments was assessed empirically. As mentioned in the HME section, a number of filaments were difficult to extrude, as they were fragile and brittle. The printability was assessed by attempting to load the filament into the printer. As expected, the most fragile and brittle filaments were not possible to print, as they would break apart due to forces exerted by the gears that feed the filament into the printhead, and thus were deemed unprintable. In many cases, a visual and tactile assessment of an extruded filament is sufficient to evaluate whether or not the filament is printable. In Fig. S3 images of filament samples are depicted. Filaments that were deemed printable were those filaments that could be printed directly, or only required minor adjustments of, for example, the printhead's speed. Table 6 summarizes the printability of all samples produced of each mesoporous material. For a summary of the printability of all samples along with their composition, extrusion temperature and mechanical properties, see Table S1. For MMC, 7 of the 19 samples were printable, whereas for MCM-41 13 of the 19 samples were printable.

**Table 6:** Printability assessment for MMC and MCM-41 filament samples.

Run Order	MMC filament Printability	MCM-41 filament Printability
1	Yes	No
2	Yes	Yes
3	No	Yes
4	Yes	Yes
5	Yes	No
6	Yes	Yes
7	No	Yes
8	No	No
9	Yes	Yes
10	No	Yes
11	No	No
12	Yes	Yes
13	No	Yes
14	No	No
15	No	Yes
16	No	Yes
17	No	No
18	No	Yes
19	No	Yes

### 3.8 Mechanical studies

Generally, a filament for FDM needs to be sufficiently elastic and rigid in order for it to be feedable via the printer's gears without breaking or getting jammed. Various mechanical properties of filaments have been previously investigated and correlated with their printability, including hardness, stiffness, resistance, maximum stress, elongation at break, and Young's modulus (Gioumouxouzis et al., 2020; Yang et al., 2021; Đuranović et al., 2021; Xu et al., 2020). Among these properties, the maximum tensile stress and Young's modulus are the most commonly used, and thus were the ones investigated in this study.

The data for Force/Stroke obtained via the tensile measurements were converted to Stress/Strain and plotted. All stress/strain curves exhibited elastic behavior without plastic deformation until the samples broke at the point of maximum stress. The maximum stress point for each sample was noted and the slope of the curve corresponding to the elastic deformation of the sample was calculated to obtain the Young's modulus. Table 7 displays these values of maximum tensile strength and Young's modulus for the filaments produced with each mesoporous material. Samples containing MMC, in general, had lower values for both maximum tensile stress and Young's modulus, compared to filaments containing MCM-41. This could be partially explained by differences in particle size of the mesoporous materials and their flowability, which in turn affect the homogeneity of the mixture in the extruder's barrel and the resulting filament.

**Table 7:** Young's modulus and maximum tensile stress values for MMC and MCM-41 filament samples.

Run Order	MMC		MCM-41	
	Young's Modulus	Max tensile stress	Young's Modulus	Max tensile stress
1	67.5	3.0	66.2	3.5
2	97.1	3.7	120.3	7.5
3	50.7	1.4	167.2	25.2
4	71.6	3.6	191.4	13.8
5	182.7	12.7	47.0	2.3
6	53.4	4.0	114.0	18.8
7	117.7	7.5	134.7	8.7
8	94.5	11.1	66.3	2.4
9	64.6	6.1	180.0	24.4
10	188.1	3.7	144.7	16.5
11	35.6	4.7	40.8	2.2
12	56.9	12.0	165.4	19.3
13	65.0	3.3	83.1	16.5
14	32.7	0.9	49.2	4.2
15	51.7	2.6	272.2	16.5
16	200.0	5.0	133.3	7.8
17	58.2	2.1	16.2	1.7
18	26.9	2.0	239.4	12.0
19	53.5	5.7	180.4	25.4

### 3.9 Factorial analysis and PCA

To explore the potential effect of the studied factors on the mechanical properties (maximum tensile stress and Young's modulus) of the filaments, a factorial regression for each response was performed. The analysis was conducted with a stepwise selection of terms in order to automatically determine the model that better fits the data. The main effects of the factors (% drug loading, % mesoporous material content in filament, plasticizer/polymer ratio, and extrusion temperature) on the two response variables as well as their two-way interactions were assessed.

**Fig. 4:**
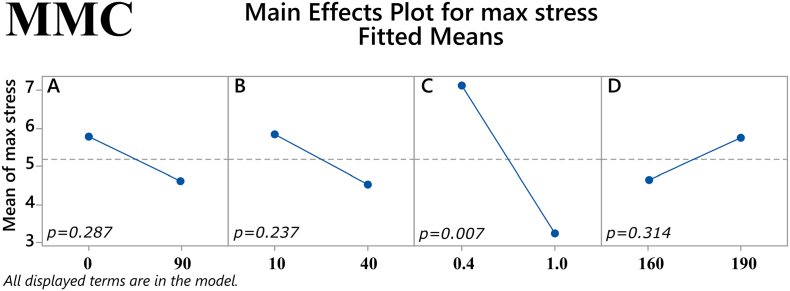
Main effects on maximum tensile stress of MMC filament samples. *(Factors A: % drug loading, B: % mesoporous material content, C: plasticizer/polymer ratio, D: extrusion temperature)*. The *p-*value for each main effect is shown in the corresponding plot.

**Fig. 5:**
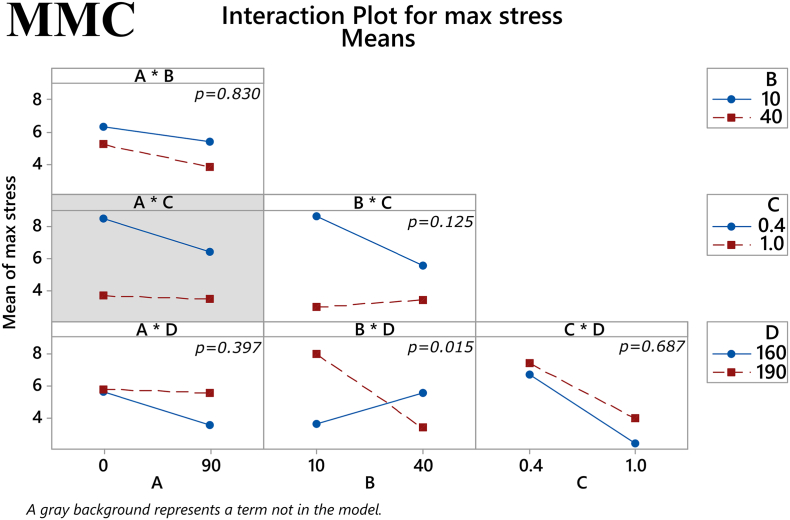
Two-way interactions on maximum tensile stress of MMC filament samples. *(Factors A: % drug loading, B: % mesoporous material content, C: plasticizer/polymer ratio, D: extrusion temperature)*. The *p-*value for each two-way interaction is shown in the corresponding plot.

**Fig. 6:**
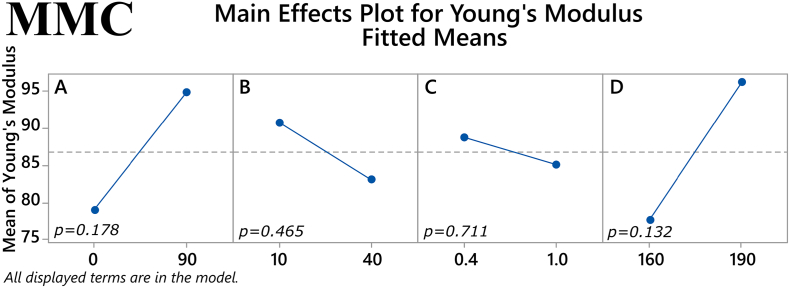
Main effects on Young's modulus of MMC filament samples. *(Factors A: % drug loading, B: % mesoporous material content, C: plasticizer/polymer ratio, D: extrusion temperature)*. The *p-*value for each main effect is shown in the corresponding plot.

**Fig. 7:**
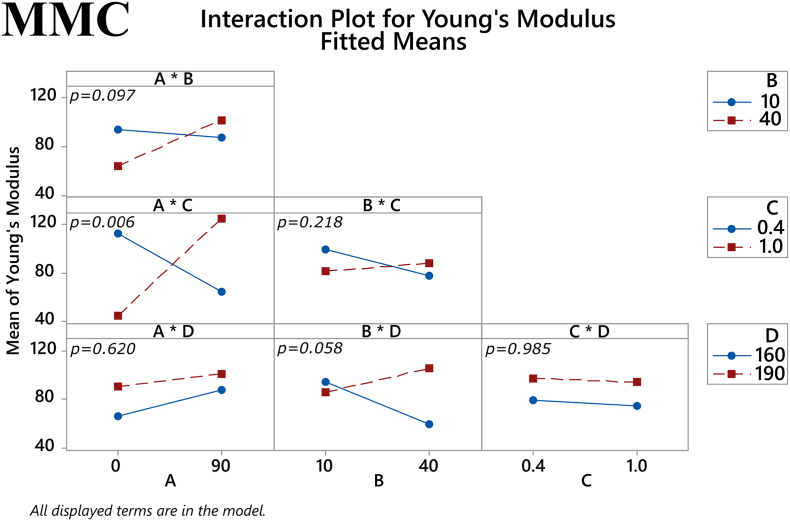
2-way interactions on Young's modulus of MMC filament samples. *(Factors A: % drug loading, B: % mesoporous material content, C: plasticizer/polymer ratio, D: extrusion temperature)*. The *p-*value for each two-way interaction is shown in the corresponding plot.

In MMC formulations, the ratio of plasticizer/polymer was revealed to have a significant main effect on maximum stress (*p* = 0.007), where a higher ratio would yield lower values for the response. The other three factors showed no statistically significant main effects, see Fig. 4. However, the two-way interaction of the factors % mesoporous material content in the filament and extrusion temperature was statistically significant (*p* = 0.015), as seen in Fig. 5. Regarding the other response examined, i.e., Young's modulus, no statistically significant main effects were found with MMC samples, see Fig. 6. Despite the lack of main effects, the two-way interaction of % drug loading and plasticizer/polymer ratio was found to be statistically significant (*p* = 0.006), and the two-way interaction of % mesoporous material content in the filament and extrusion temperature approached statistical significance (*p* = 0.058), see Fig. 7.

**Fig. 8:**
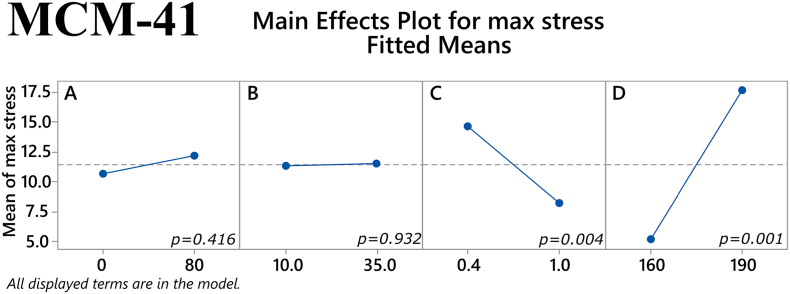
Main effects on maximum tensile stress of MCM-41 filament samples. *(Factors A: % drug loading, B: % mesoporous material content, C: plasticizer/polymer ratio, D: extrusion temperature)*. The *p-*value for each main effect is shown in the corresponding plot.

**Fig. 9:**
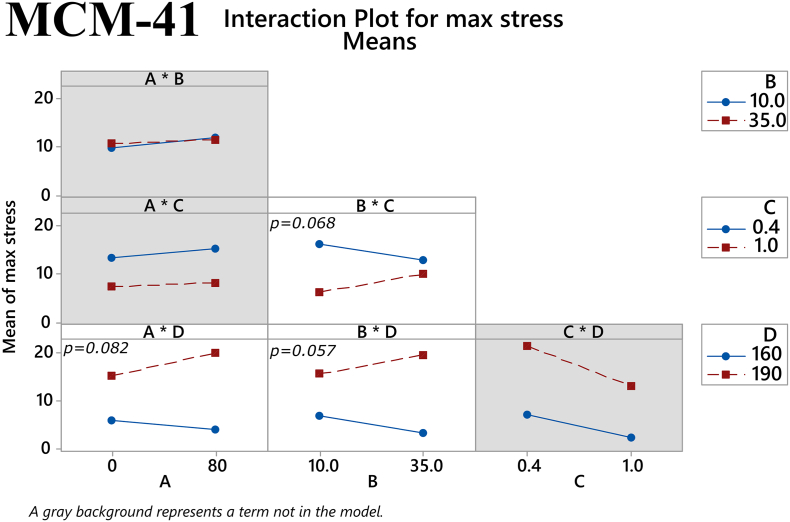
2-way interactions on maximum tensile stress of MCM-41 filament samples. *(Factors A: % drug loading, B: % mesoporous material content, C: plasticizer/polymer ratio, D: extrusion temperature)*. The *p-*value for each two-way interaction is shown in the corresponding plot.

**Fig. 10:**
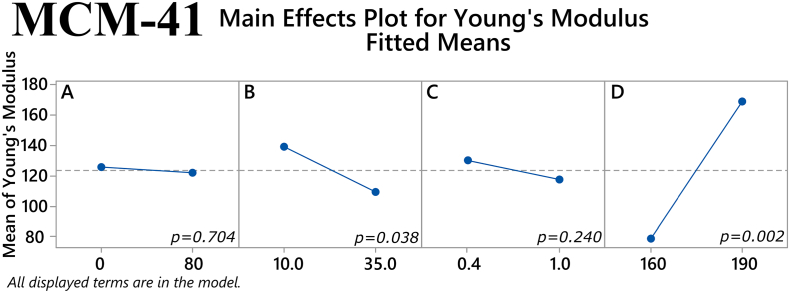
Main effects on Young's modulus of MCM-41 filament samples. *(Factors A: % drug loading, B: % mesoporous material content, C: plasticizer/polymer ratio, D: extrusion temperature)*. The *p-*value for each main effect is shown in the corresponding plot.

**Fig. 11:**
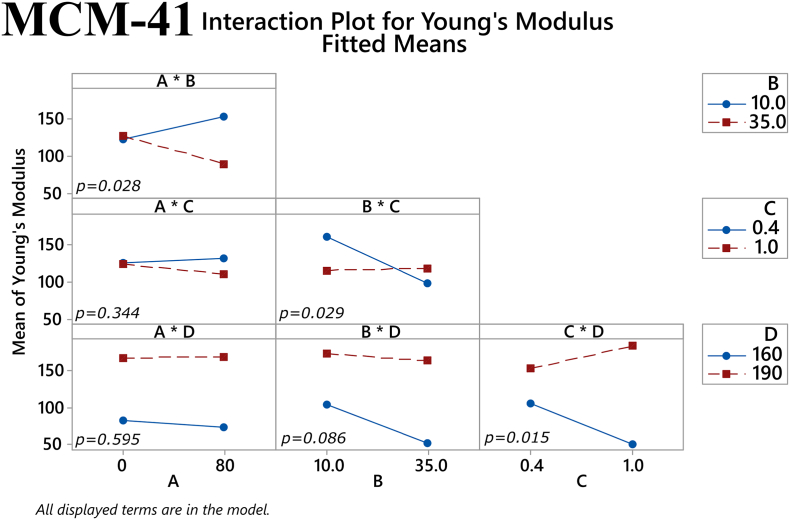
2-way interactions on Young's modulus of MCM-41 filament samples. *(Factors A: % drug loading, B: % mesoporous material content, C: plasticizer/polymer ratio, D: extrusion temperature)*. The *p-*value for each two-way interaction is shown in the corresponding plot.

The main effects of the factors on maximum tensile stress for the MCM-41 filaments are depicted in Fig. 8. The plasticizer/polymer ratio and temperature both had a statistically significant effect on this response (*p* = 0.004 and *p* < 0.001, respectively). The former suggests that although the addition of plasticizer can improve the mechanical properties of a filament, the thermoplastic polymer has a greater influence on these properties and thus a high enough percentage of it is needed in the total mixture. The latter reaffirms the notion that higher temperatures used in HME will result in better overall filament properties. Furthermore, one two-way interaction the factors was also found to approach statistical significance, namely the % mesoporous material content in the filament and extrusion temperature (*p* = 0.057), see Fig. 9. Regarding Young's modulus, both the extrusion temperature and the % mesoporous material content in the filament had a statistically significant main effect on the response (*p* = 0.002 and *p* = 0.038, respectively), as shown in Fig. 10. Finally, in Fig. 11 it can be seen that three of the factors' two-way interactions also exhibited considerable effects on the Young's modulus, specifically the factor pairs % drug loading and % mesoporous material content in the filament (*p* = 0.028), % mesoporous material content in the filament and plasticizer/polymer ratio (*p* = 0.029), and finally plasticizer/polymer ratio and extrusion temperature (*p* = 0.015).

**Fig. 12:**
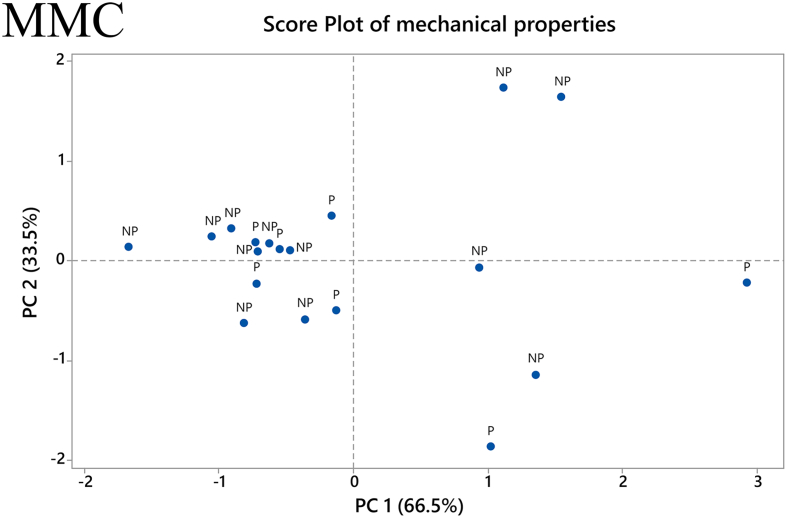
Score plot of the mechanical properties of MMC filaments. *P: Printable filaments, NP: Non-printable filaments.*

A PCA was performed to improve the visualization of the printability results and show how they may be correlated to the mechanical properties of the filaments. For the MMC samples, the two principal components, PC 1 and PC 2, are used for the explanation of 66.5% and 33.5%, respectively, of the acquired data. The contribution of each mechanical property variable, i.e., Young's modulus and maximum stress, for each PC was equal. Fig. 12 depicts the score plot for the MMC samples. The data is spread in a way that no particular grouping can be ascertained regarding the printability of the samples. There is a large accumulation of data points on the left side of the graph, with lower PC 1 scores, but because it is a mixture of printable and non-printable samples, this only informs us that the majority of the samples have low values of Young's modulus and maximum tensile stress.

**Fig. 13:**
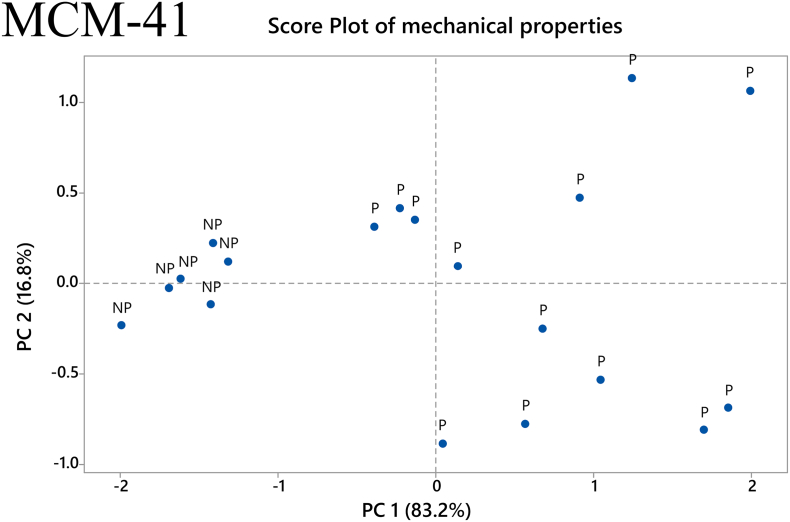
Score plot of the mechanical properties of MCM-41 filaments. *P: Printable filaments, NP: Non-printable filaments.*

The same procedure was followed in the PCA analysis of the MCM-41 filaments. In this case, the two principal components PC 1 and PC 2 explained 83.2% and 16.8%, respectively, of the data. The maximum tensile stress and Young's modulus contribute equally to each PC in this PCA as well. The score plot for MCM-41 formulations is presented in Fig. 13. Interestingly, a well-defined grouping of samples is observed regarding printability. All the non-printable samples appear on the left side of the panel, for low scores of PC 1. This suggests that the combination of low values of maximum tensile stress and Young's modulus results in non-printable filaments containing MCM-41. Moreover, it is noted that the majority of printable samples lie on the right side of the graph. This indicates that for a filament containing MCM-41, higher values for both of the mechanical properties examined would be required. This difference observed between the samples containing different mesoporous materials could once again be attributed to their difference in particle size and flowability within the extruder's barrel. Consequently, MMC filaments can be deemed less “predictable” compared to the ones containing MCM-41 in terms of a printability assessment based on their mechanical properties.

The relationship and possible correlation between the printability of a filament and one or more of the mechanical properties of a filament has been the focus of a number of studies, although a clear consensus does not emerge. Gioumouxouzis et al. suggested that printability increases when the elastic modulus decreases and hardness increases (Gioumouxouzis et al., 2018). More specifically, it was proposed that printable filaments had a ratio of elastic modulus/hardness <21 (Gioumouxouzis et al., 2020). In another study, printability was found to depend on several rheological and mechanical properties of the filaments, including shear-thinning and elastic modulus, where an increase in these properties improved the processability of the filaments (Ilyés et al., 2019). Tabriz et al. examined the printability of various pharmaceutical grade polymers in relation to the mechanical properties of their filaments and found that the maximum tensile stress, elongation at break and Young's modulus were found to have an effect on the printability (Tabriz et al., 2021). Of these three properties, maximum tensile stress was shown to be the most important for predicting printability, where a value of 15 to 20 MPa was identified as the threshold above which filaments were printable. However, another study of the mechanical properties of filaments concluded that the maximum displacement and force in the three-point bending test of filaments, but not the maximum tensile stress, had a clear correlation with printability (Đuranović et al., 2021). The discrepancies in the relationship between printability and the mechanical properties of filaments in these studies suggests that printability depends on several factors, and is likely highly dependent on the formulations of the filaments.

Returning to the results from the present study, we observe that a consistent relationship between the investigated mechanical properties and printability could not be found with both the MMC-containing and MCM-41-containing filaments. While the values for maximum stress and Young's modulus for the few printable MMC-containing samples did not reveal any trend, the values for maximum stress and Young's modulus for printable MCM-41 formulations were > 7.5 MPa and > 83 MPa, respectively. In order to optimize formulations with mechanical properties exceeding these values we can turn to the results of the factorial analysis to see which factors showed statistically significant effect on the respective mechanical property. For MCM-41 formulations, both the plasticizer/polymer ratio and extrusion temperature had statistically significant main effects on the maximum stress, while the % mesoporous material content and extrusion temperature had statistically significant main effects on the Young's modulus. Additionally, two-way interactions of % drug loading and % mesoporous material content, plasticizer/polymer ratio and % mesoporous material content, and plasticizer/polymer ratio and extrusion temperature were also found to have a statistically significant effect on the Young's modulus. Of course, there were also factors that were found to have statistically significant effect on the mechanical properties of the MMC formulations, but it is difficult to use the information from the factorial analysis to optimize MMC-containing formulations since the PCA analysis did not reveal any discernable pattern relating the printability of the MMC-containing filaments to the measured mechanical properties. The lack of observed trend with the MMC-containing samples can in large part be attributed to the filaments' inconsistencies and imperfections. The heterogeneous nature of the filaments can in turn be attributed to the inclusion of the non-melting mesoporous materials in the formulations, which was particularly apparent with MMC, see Fig. S3. Very few studies have investigated the effect of non-melting components in filaments, however, a study incorporating talc as an inert filler was used to improve the printability of a poorly printable polymer. (Yang et al., 2021). It was observed that adding talc increases strength, i.e., the maximum stress before breaking, but also reduces the flexibility of the filaments, i.e., the maximum strain or elongation at breaking. In essence the filament becomes more brittle with larger proportions of the inert filler. A range of maximum stress values of 2.3–8.5 MPa was deemed to be optimal for printability, depending on the polymer used. An additional observation is that higher proportions of talc reduced the material cohesion, resulting in poorer prints due to high talc content. We can observe similar effects with increased mesoporous carrier content. However, in our situation we are trying to maximize the proportion of MMC/MCM-41, acting as the drug carrier, in the filament and trying to find filament components ratios (polymer, plasticizers) and processing parameters (extrusion temperatures) that will improve printability.

Although the current study focused on Young's modulus and maximum stress of the filaments, there may be other properties that could be correlated to the printability of filaments containing mesoporous materials. Not only does a filament have to be able to withstand the mechanical forces imposed by the feeding gears, but the feedstock should possess the rheological properties upon melting in the printhead that allow it to flow unimpeded through the printing nozzle. Therefore, mechanical properties like hardness and rheological properties like shear-thinning would be interesting to investigate in future studies. For example, indentation tests could be used to assess the hardness of the filaments. However, it is worthwhile to point out that the imperfections in the filaments produced with mesoporous materials would likely make such an investigation difficult as local variations can produce results that are not necessarily representative of the entire formulation. Yet another challenge imposed by the presence of the non-melting mesoporous particles is the potential for aggregation and subsequent clogging of the nozzle.

## 4 Conclusions

In the current work, the effect of four processing factors on the mechanical properties of extruded filaments containing mesoporous materials was investigated with the aim of determining, the relationship between the mechanical properties and the filaments' printability. A two-level full-factorial experimental design was employed to set up the experiments and statistically assess the results. A correlation was found between non-printable filaments of MCM-41 and their mechanical properties where printable filaments had a maximum stress >7.5 MPa and a Young's modulus >83 MPa. On the other hand, MMC filaments did not exhibit any correlation, which was in large part attributed to the filaments' inconsistencies and imperfections. Finally, both mesoporous materials displayed a thermal protective feature, as the onset and/or the rate of the thermal degradation of the thermolabile drug was shifted to higher temperatures post-loading. This highlights the potential capability of such a system to be implemented for thermosensitive drugs in FDM applications.

## Funding

This work is conducted within the Additive Manufacturing for the Life Sciences Competence Center (AM4Life). The authors gratefully acknowledge financial support from Sweden's Innovation Agency VINNOVA (Grant no: 2019-00029) and the Swedish Science Council (Grant no: 2019-03729).

## Author contribution

Conceptualization, C.S.K., K.W. and M.S.; investigation, C.S.K.; writing—original draft preparation, C.S.K.; writing—review and editing, C.S.K., K.W. and M.S.; visualization, C.S.K.; supervision, K.W. and M.S.; funding acquisition, M.S. All authors have read and agreed to the published version of the manuscript.

## Declaration of Competing Interest

The authors declare the following financial interests/personal relationships which may be considered as potential competing interests: Maria Stromme reports financial support was provided by Swedish Research Council. Maria Stromme reports financial support was provided by Sweden's Innovation Agency.

## Data Availability

Data will be made available on request.

## Appendix A Supplementary data

Supplementary material Image 1
