# Supplementary material for: Processability of mesoporous materials in fused deposition modeling for drug delivery of a model thermolabile drug
Source: Int J Pharm X. 2022 Dec 17;5:100149. doi: 10.1016/j.ijpx.2022.100149 (PMC9804103; doi:10.1016/j.ijpx.2022.100149)
Supplement: Supplementary file 1 — Supplementary material [file mmc1.docx]

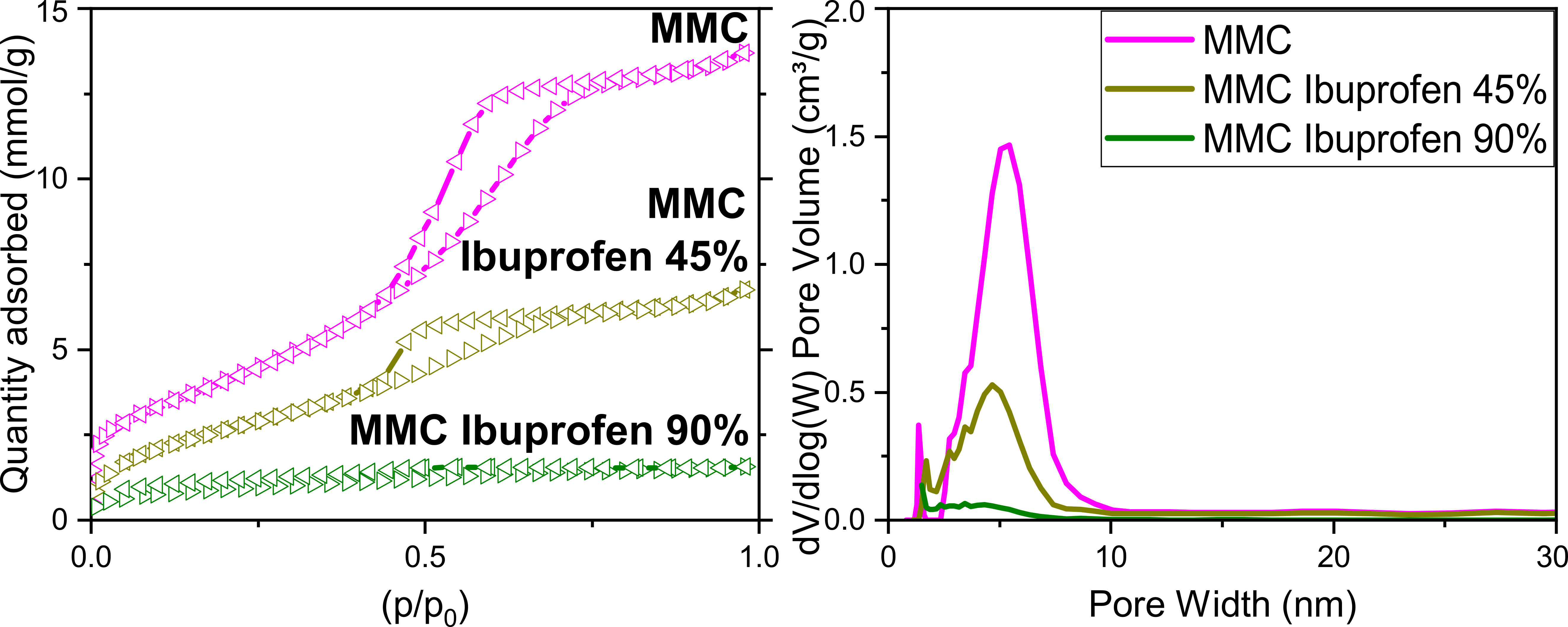


Figure S1. Nitrogen gas sorption isotherms for unloaded and drug-loaded MMC samples and their pore size distributions.


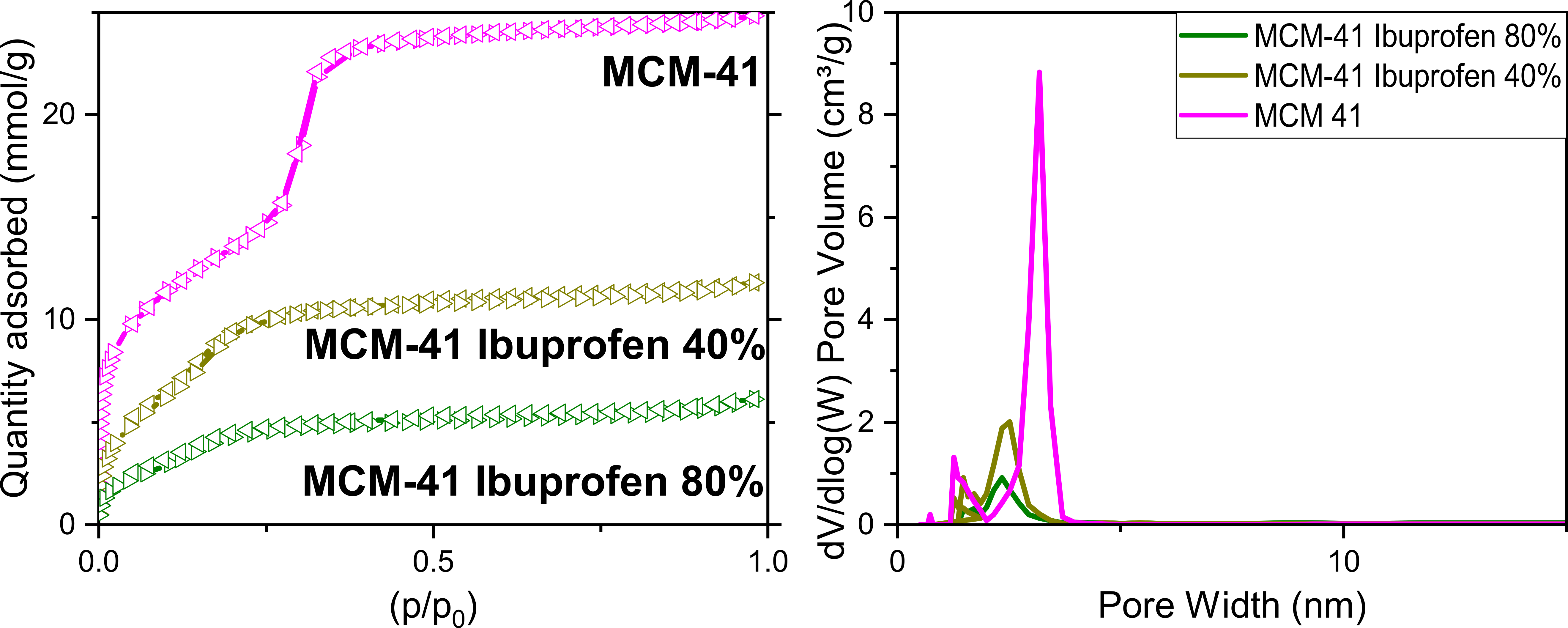


Figure S2. Nitrogen gas sorption isotherms for unloaded and drug-loaded MCM-41 samples and their pore size distributions.

**
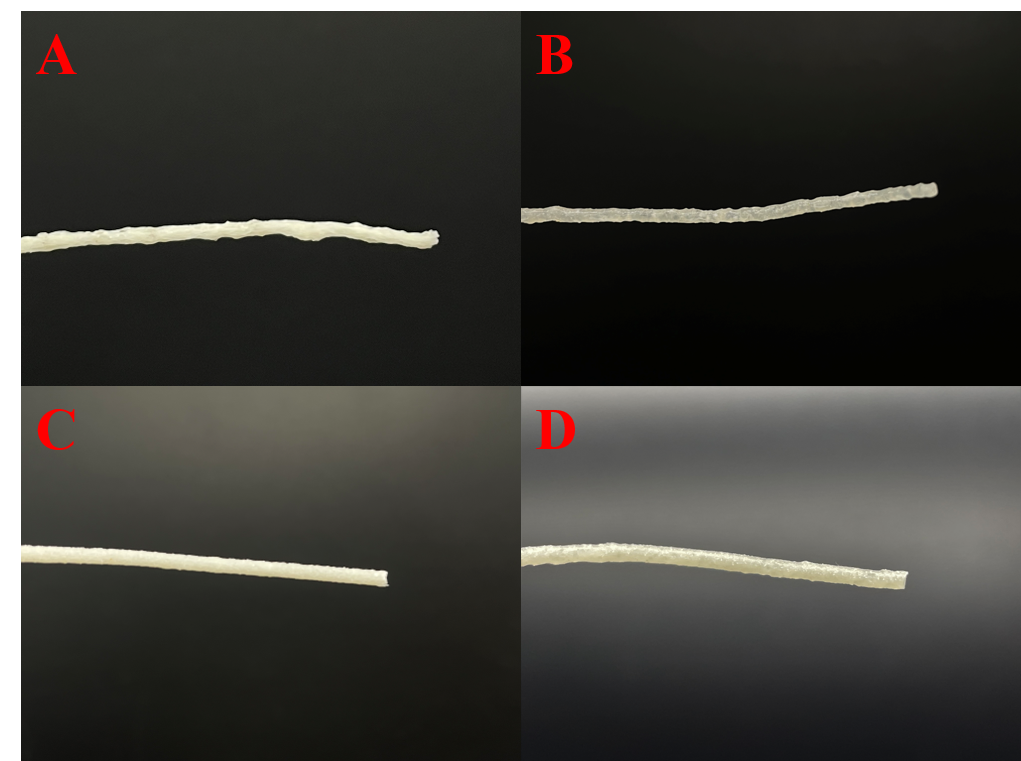
**

Figure S3. Images of filament samples (A) MMC Run 13, (B) MMC Run 10, (C) MMC Run 12, (D) MCM-41 Run 2. Filaments A and B, which were found to be unprintable, have visible morphological imperfections and a rough surface compared to C and D, which were found to be printable. Under further tactile inspection, filament samples A and B would also easily break under minimal force, revealing their brittleness.


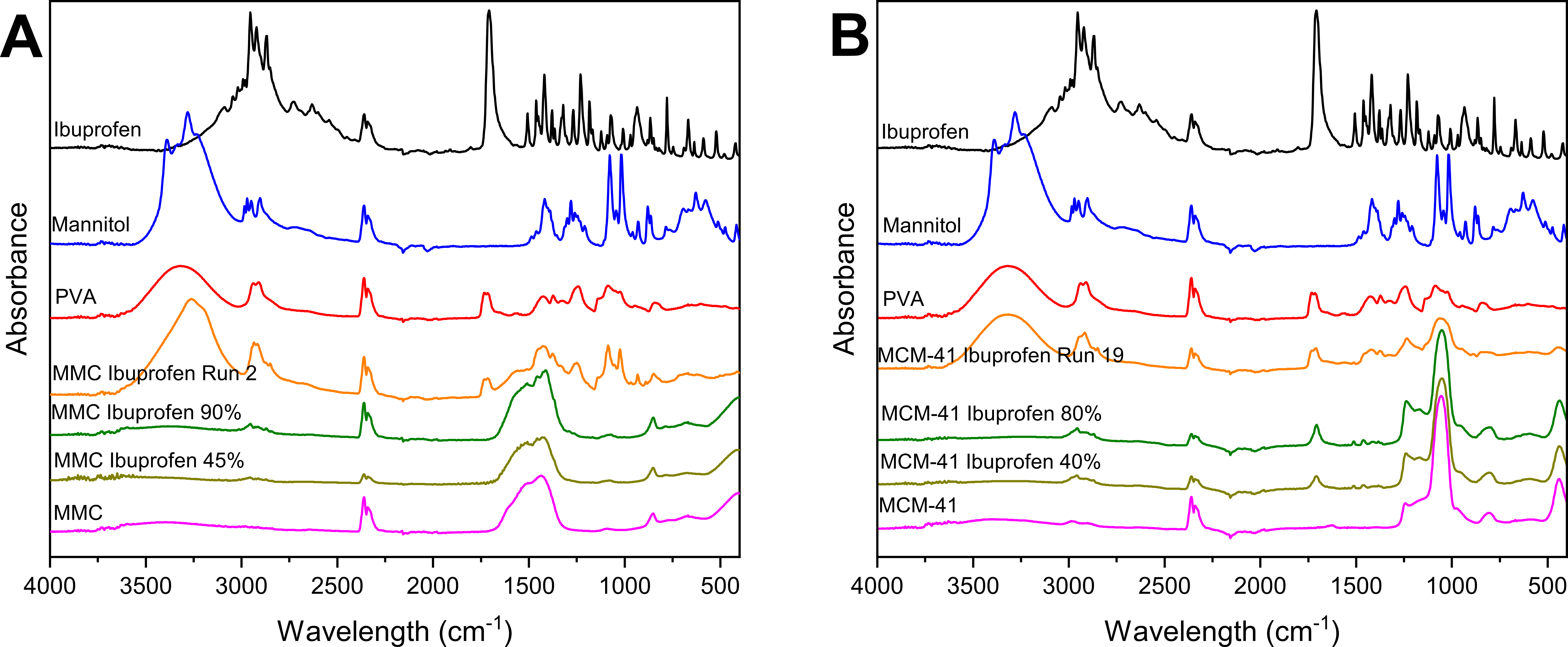


Figure S4. ATR-FTIR spectra of Ibuprofen, the individual component powders of the filaments, and sample filaments MMC Run 2 and MCM-41 Run 19 for (A) MMC-containing samples, and (B) MCM-41-containing samples.


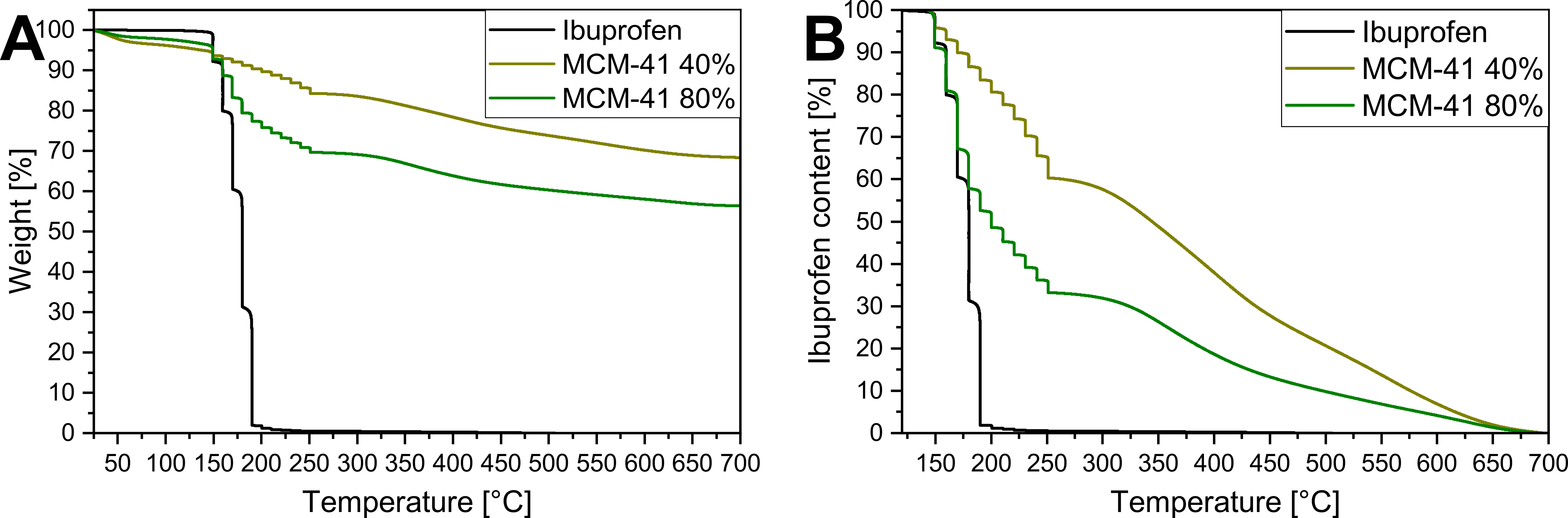


Figure S5. (A) TGA thermograms of ibuprofen and MCM-41 drug-loaded samples. (B) TGA thermograms of ibuprofen and MCM-41 drug-loaded samples normalized to the ibuprofen content. In these experiments the temperature was increased at 10 ºC/min until 150 ºC at which point the temperature was increased in discrete steps of 10 ºC and held at this temperature for 20 min until reaching 250 ºC, at which point the temperature was increased at a rate of 10 º/min until 700 ºC.

Table S1. Summary of the composition of filaments for each experimental run, as well as extrusion temperature, mechanical properties and printability results.

|  | **MMC** | | | | | | | | **MCM-41** | | | | | | | |
| --- | --- | --- | --- | --- | --- | --- | --- | --- | --- | --- | --- | --- | --- | --- | --- | --- |
| **Run Order** | **% Drug Loading** | **% Meso-**  **porous** | **% Plasticizer** | **% Polymer** | **Temp (ºC)** | **Young’s Modulus** | **Max tensile stress** | **Printability** | **% Drug Loading** | **% Meso-**  **porous** | **% Plasticizer** | **% Polymer** | **Temp (ºC)** | **Young’s Modulus** | **Max tensile stress** | **Printability** |
| 1 | 0 | 40 | 17 | 43 | 190 | 67.5 | 3.0 | Yes | 0 | 35.0 | 32.5 | 32.5 | 160 | 66.2 | 3.5 | No |
| 2 | 90 | 40 | 17 | 43 | 190 | 97.1 | 3.7 | Yes | 40 | 22.5 | 32 | 45.5 | 175 | 120.3 | 7.5 | Yes |
| 3 | 45 | 25 | 31 | 44 | 175 | 50.7 | 1.4 | No | 80 | 10.0 | 26 | 64 | 190 | 167.2 | 25.2 | Yes |
| 4 | 0 | 40 | 30 | 30 | 190 | 71.6 | 3.6 | Yes | 0 | 10.0 | 26 | 64 | 160 | 191.4 | 13.8 | Yes |
| 5 | 0 | 10 | 26 | 64 | 190 | 182.7 | 12.7 | Yes | 80 | 35.0 | 32.5 | 32.5 | 160 | 47.0 | 2.3 | No |
| 6 | 90 | 10 | 26 | 64 | 160 | 53.4 | 4.0 | Yes | 0 | 35.0 | 18.5 | 46.5 | 190 | 114.0 | 18.8 | Yes |
| 7 | 0 | 10 | 26 | 64 | 160 | 117.7 | 7.5 | No | 80 | 10.0 | 26 | 64 | 160 | 134.7 | 8.7 | Yes |
| 8 | 0 | 40 | 17 | 43 | 160 | 94.5 | 11.1 | No | 80 | 10.0 | 45 | 45 | 160 | 66.3 | 2.4 | No |
| 9 | 90 | 40 | 17 | 43 | 160 | 64.6 | 6.1 | Yes | 40 | 22.5 | 32 | 45.5 | 175 | 180.0 | 24.4 | Yes |
| 10 | 90 | 10 | 45 | 45 | 160 | 188.1 | 3.7 | No | 0 | 10.0 | 26 | 64 | 190 | 144.7 | 16.5 | Yes |
| 11 | 0 | 40 | 30 | 30 | 160 | 35.6 | 4.7 | No | 80 | 35.0 | 18.5 | 46.5 | 160 | 40.8 | 2.2 | No |
| 12 | 90 | 10 | 26 | 64 | 190 | 56.9 | 12.0 | Yes | 40 | 22.5 | 32 | 45.5 | 175 | 165.4 | 19.3 | Yes |
| 13 | 90 | 10 | 45 | 45 | 190 | 65.0 | 3.3 | No | 80 | 35.0 | 32.5 | 32.5 | 190 | 83.1 | 16.5 | Yes |
| 14 | 0 | 10 | 45 | 45 | 160 | 32.7 | 0.9 | No | 0 | 35.0 | 18.5 | 46.5 | 160 | 49.2 | 4.2 | No |
| 15 | 45 | 25 | 31 | 44 | 175 | 51.7 | 2.6 | No | 0 | 35.0 | 32.5 | 32.5 | 190 | 272.2 | 16.5 | Yes |
| 16 | 90 | 40 | 30 | 30 | 190 | 200.0 | 5.0 | No | 0 | 10.0 | 45 | 45 | 190 | 133.3 | 7.8 | Yes |
| 17 | 90 | 40 | 30 | 30 | 160 | 58.2 | 2.1 | No | 0 | 10.0 | 45 | 45 | 160 | 16.2 | 1.7 | No |
| 18 | 45 | 25 | 31 | 44 | 175 | 26.9 | 2.0 | No | 80 | 10.0 | 45 | 45 | 190 | 239.4 | 12.0 | Yes |
| 19 | 0 | 10 | 45 | 45 | 190 | 53.5 | 5.7 | No | 80 | 35.0 | 18.5 | 46.5 | 190 | 180.4 | 25.4 | Yes |

Table S2. MMC percentage w/w in theory, in theory accounting for the presence of drug, and in practice calculated via TGA.

| **Run Order** | **MMC content in mixture theoretical (%)** | **MMC content in mixture theoretical accounting for drug (%)** | **MMC content calculated by TGA (%)** |
| --- | --- | --- | --- |
| 1 | 40 | 40.00 | 37.11 |
| 2 | 40 | 28.24 | 25.03 |
| 3 | 25 | 20.93 | 21.80 |
| 4 | 40 | 40.00 | 32.56 |
| 5 | 10 | 10.00 | 8.99 |
| 6 | 10 | 7.06 | 7.58 |
| 7 | 10 | 10.00 | 9.15 |
| 8 | 40 | 40.00 | 24.0 |
| 9 | 40 | 28.24 | 24.50 |
| 10 | 10 | 7.06 | 6.01 |
| 11 | 40 | 40.00 | 29.1 |
| 12 | 10 | 7.06 | 6.53 |
| 13 | 10 | 7.06 | 4.94 |
| 14 | 10 | 10.00 | 9.52 |
| 15 | 25 | 20.93 | 17.88 |
| 16 | 40 | 28.24 | 26.63 |
| 17 | 40 | 28.24 | 25.64 |
| 18 | 25 | 20.93 | 20.11 |
| 19 | 10 | 10.00 | 8.64 |

Table S3. MCM-41 percentage w/w % in theory, in theory accounting for the presence of drug, and in practice calculated via TGA.

| **Run Order** | **MCM-41 content in mixture theoretical (%)** | **MCM-41 content in mixture theoretical accounting for drug (%)** | **MCM-41 content calculated by TGA (%)** |
| --- | --- | --- | --- |
| 1 | 35 | 35 | 27.9 |
| 2 | 22.5 | 17.08 | 15.43 |
| 3 | 10 | 6.26 | 5.77 |
| 4 | 10 | 10 | 8.3 |
| 5 | 35 | 21.91 | 18.7 |
| 6 | 35 | 35.00 | 28.68 |
| 7 | 10 | 6.26 | 5.85 |
| 8 | 10 | 6.26 | 5.91 |
| 9 | 22.5 | 17.08 | 11.07 |
| 10 | 10 | 10.00 | 7.98 |
| 11 | 35 | 21.91 | 17.9 |
| 12 | 22.5 | 17.08 | 16.32 |
| 13 | 35 | 21.91 | 15.3 |
| 14 | 35 | 35.00 | 30.18 |
| 15 | 35 | 35.00 | 25.64 |
| 16 | 10 | 10.00 | 9.49 |
| 17 | 10 | 10.00 | 8.85 |
| 18 | 10 | 6.26 | 4.81 |
| 19 | 35 | 21.91 | 17.57 |
